# A Network Approach for the Study of Drug Prescriptions: Analysis of Administrative Records from a Local Health Unit (ASL TO4, Regione Piemonte, Italy)

**DOI:** 10.3390/ijerph18094859

**Published:** 2021-05-02

**Authors:** Gianluca Miglio, Lara Basso, Lucrezia G. Armando, Sara Traina, Elisa Benetti, Abdoulaye Diarassouba, Raffaella Baroetto Parisi, Mariangela Esiliato, Cristina Rolando, Elisa Remani, Clara Cena

**Affiliations:** 1 Dipartimento di Scienza e Tecnologia del Farmaco, Università degli Studi di Torino, Via Pietro Giuria 9, 10125 Turin, Italy; lara.basso@edu.unito.it (L.B.); lucrezia.armando@edu.unito.it (L.G.A.); sara.traina@edu.unito.it (S.T.); elisa.benetti@unito.it (E.B.); clara.cena@unito.it (C.C.); 2 Centro di Competenza sul Calcolo Scientifico C3S, Università degli Studi di Torino, Corso Svizzera 185, 10149 Turin, Italy; 3 Struttura Complessa Farmacia Territoriale ASL TO4, Regione Piemonte, Via Po 11, 10034 Chivasso, Italy; adiarassouba@aslto4.piemonte.it (A.D.); rbaroettoparisi@aslto4.piemonte.it (R.B.P.); mesiliato@aslto4.piemonte.it (M.E.); crolando@aslto4.piemonte.it (C.R.); elisa.remani@edu.unito.it (E.R.)

**Keywords:** drug prescriptions, networks, pattern recognition

## Abstract

In a Drug Prescription Network (DPN), each drug is represented as a node and two drugs co-prescribed to the same patient are represented as an edge linking the nodes. The use of DPNs is a novel approach that has been proposed as a means to study the complexity of drug prescription. The aim of this study is to demonstrate the analytical power of the DPN-based approach when it is applied to the analysis of administrative data. Drug prescription data that were collected at a local health unit (ASL TO4, Regione Piemonte, Italy), over a 12-month period (July 2018–June 2019), were used to create several DPNs that correspond to the five levels of the Anatomical Therapeutic Chemical classification system. A total of 5,431,335 drugs prescribed to 361,574 patients (age 0–100 years; 54.7% females) were analysed. As indicated by our results, the DPNs were dense networks, with giant components that contain all nodes. The disassortative mixing of node degrees was observed, which implies that non-random connectivity exists in the networks. Network-based methods have proven to be a flexible and efficient approach to the analysis of administrative data on drug prescription.

## 1. Introduction

Drug prescription analysis is attaining resonance for multiple reasons. Firstly, the growing number of older adults means that there is an increased need to monitor comorbidity and poly-therapeutic regimens [1,2]. Secondly, the analysis of prescription patterns may be extremely useful for identifying potential inappropriate prescriptions, adverse drug events and potential drug–drug interactions, as well as for assessing epidemiological models and medication adherence on a large scale [3]. The analysis of medical records could also improve the delivery of care in healthcare settings, as it may be able to provide predictive and prescriptive knowledge. Lastly, research into drug prescription patterns may also be a way to rationalise and possibly prevent hospitalisation, and therefore reduce healthcare costs [4].

The increase in large-scale drug (co)prescription data collected by healthcare systems provides major challenges to the healthcare providers and regulators who are seeking to recognise patterns or trends from this massive amount of data. A data mining technique could be used to analyse and thus convert the collected raw data into specific information and knowledge desired by end-users. Data on drug prescriptions can be analysed by adopting inferential statistics [5], and can also be studied using network models. Network (graph) theory has been used to explore graphs since the 18th century [6]. A network consists of nodes (vertices), which represent the entities of the system. Pairs of nodes are joined by links (edges), which represent a particular kind of interconnection between those entities. In recent decades, research into complex networks has exploded thanks to the great analytical power that network models have to describe real-world applications [7,8,9]. Network models have also been used for the study of drug prescriptions, and the notion of a Drug Prescription Network (DPN) was introduced in two previous studies [10,11]. Each prescription in a DPN is represented by a node, while a pair of co-prescriptions to the same patient is represented by an edge linking the two nodes. Although the use of DPNs is still in its infancy, it has been demonstrated to be a valid means with which to highlight a number of network features, from the local scale (nodes and edges) to the global scale (the overall network).

The aim of this study is to expand upon previous findings on DPNs as a method for the study of drug prescriptions. In particular, concepts of the network theory were adopted to recognise patterns in a large dataset of (co)prescriptions.

## 2. Materials and Methods

### 2.1. Data

Drugs prescribed by general practitioners (GPs) operating in the Italian National Health Service setting in the area north of the city of Turin were considered in this study. The source of data was the local health unit (LHU; ASL TO4, Regione Piemonte, Italy) database and the work was carried out in compliance with the General Data Protection Regulation-EU 2016/679. In particular, the study was focused on the implementation of a methodological approach to describe the complexity of drug co-prescription through the adoption of network models, and the data were treated in full accordance with current privacy legislation.

### 2.2. Drug Prescription Networks

**Figure 1:**
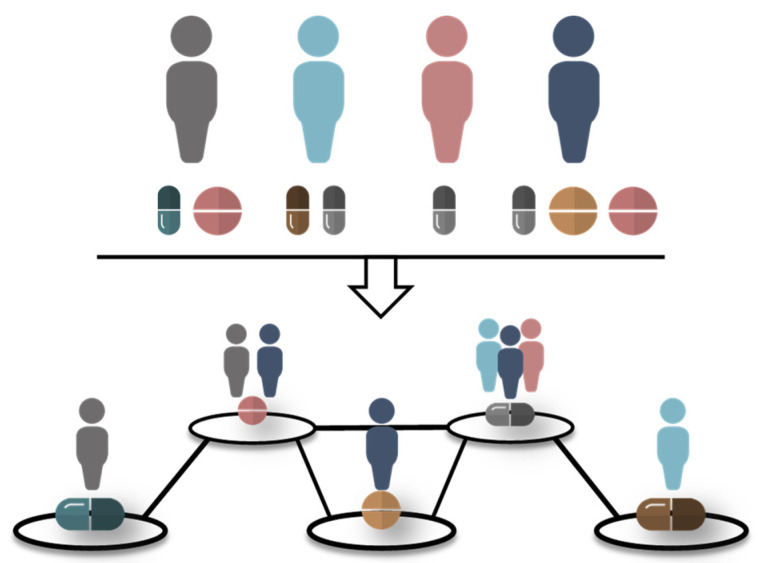
Representation of a drug prescription network. Data on drug (co)prescriptions were analysed to create drug prescription networks that correspond to the five levels of the Anatomical Therapeutic Chemical (ATC) classification system. Nodes and edges in these networks were the single ATCs and ATC–ATC pairs found in the original dataset, respectively.

**Figure A1:**
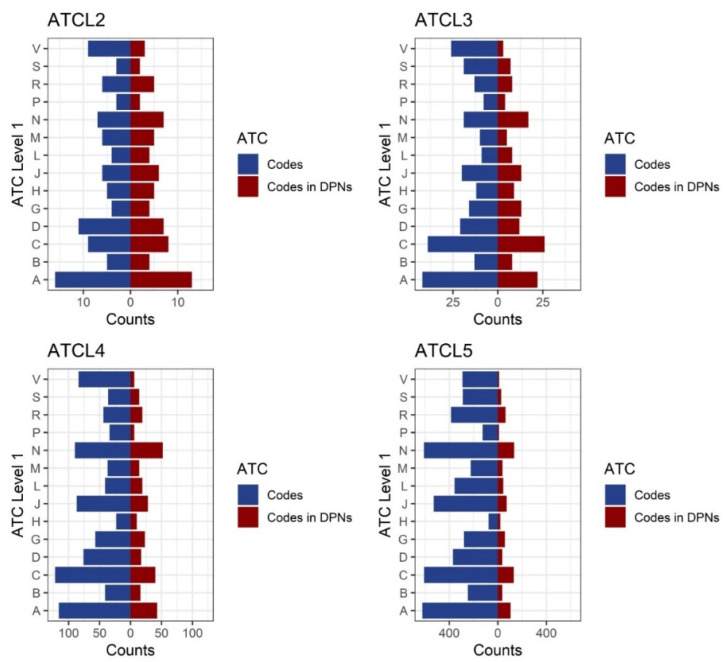
The ATC classification system. The ATC classification system consists of five levels and several subcategories nested within broader parent categories. The resulting structural organization of the ATC codes is shown in the figure. In particular, the ATC codes were counted both in the overall system (blue columns) and, for comparison, in the DPNs (dark red columns) examined in this study.

Data were provided in the form of DPNs. Each entry in the dataset was associated with a unique code from the Anatomical Therapeutic Chemical (ATC) classification system [12]. In particular, the ATC classification system consists of five levels (L), with subcategories nested within broader parent categories (Figure A1). Data on prescriptions of each ATC*_i,L_* (where i refers to drug classification at the given ATC level L) and co-prescriptions of pairs ATC*_i,L_*–ATC*_j,L_* (with i ≠ j) were considered, generating 5 networks (DPN-ATCL1–DPN-ATCL5). In these networks, the nodes were the ATC*_i,L_*s found in the dataset and represented the number of patient(s) ($P_{i}$) prescribed with drug i within ATC-level L. The edges were the pairs ATC*_i,L_*–ATC*_j,L_*s and represented the number of patient(s) ($C_{ij}$) co-prescribed with the drug pairs i and j within ATC-level L (Figure 1).

### 2.3. Analysis of Drug Prescription Networks

Several statistical and network measures were used to characterise the structure of the DPNs, including the degree (k), the betweenness centrality (b), the closeness centrality (Cl) of a node and their average values relative to a network ($\bar{k}$, $\bar{b}$ and $\bar{Cl}$); the density of the network (d), the assortativity coefficient (r) [13,14], the Pearson’s correlation coefficient for binary variables (*ϕ*) [11,15] and the Euclidean distance between graphs [16] were also determined. In addition, dendrograms, built using the ward. D2 linkage [17], were drawn to identify distinct clusters within the distance matrices (Appendix A).

### 2.4. Software

All analyses were performed using the R statistical and programming language (version 3.2.2; https://cran.r-project.org/; accessed on 12 February 2020) in combination with Cytoscape (version 3.7.2; https://cytoscape.org/; accessed on 12 February 2020). In addition, several add-on packages (and their dependencies) were used: circlize, ComplexHeatmap, ggplot2, ggpubr, igraph, httr, pdist, philentropy, RColorBrewer, RCy3 [18], RJSONIO.

## 3. Results

### 3.1. Study Population

**Figure 2:**
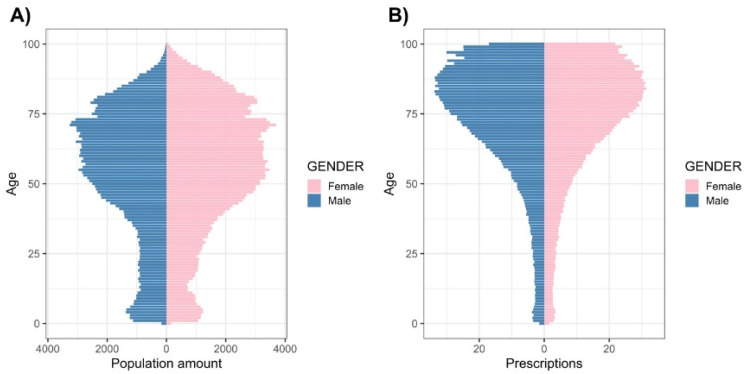
Study population. A total of 5431,335 entries/drugs prescribed to 361,574 patients were analysed in this study. Panel (**A**) shows age and sex structures of the study population. Panel (**B**) shows relative prescriptions (all prescriptions/patient) classified by age and gender.

**Figure A2:**
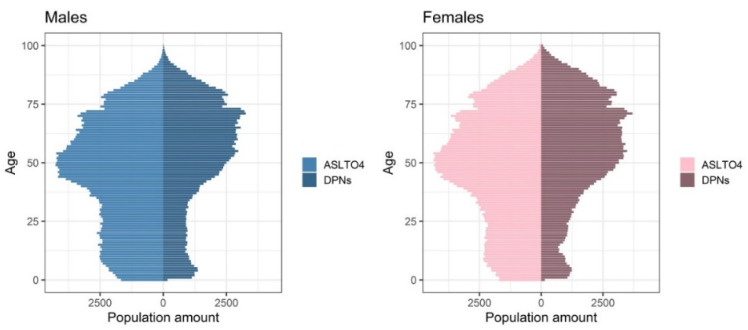
Overall and study population. Age and sex structures of the overall population (ASL TO4) and study population (DPNs) are shown.

A network-based approach was used to analyse a large dataset created from the database at an LHU (ASL TO4) in Regione Piemonte (Italy). A subset of the database containing 5,434,536 records/drugs prescribed to 361,914 patients over a 12-month period (from July 2018 to June 2019) was initially considered. Patient birth year was adopted, within the 1919–2019 range, as inclusion criteria. After excluding incomplete and/or ambiguous entries, the resulting dataset consisted of 5,431,335 entries/drugs prescribed to 361,574 patients. Therefore, this study population is a subset of the overall population (Figure A2). As shown in Figure 2, 54.7% of the patients in the study population were females. Moreover, 11.9% (6.1% and 5.8%, for males and females, respectively), 50.0% (22.4% and 27.6%) and 38.1% (16.8% and 21.3%) were of 0–21, 22–64 and ≥65 years of age, respectively. As shown in Figure 2B, 53.7% of overall drug prescriptions were attributable to female patients. In addition, 2.4% (1.3% and 1.1%), 30.1% (14.2% and 15.9%) and 67.5% (30.8% and 36.7%) of the drugs were prescribed to patients in the age ranges 0–21, 22–64 and ≥65, respectively.

Given the minimal restrictions to the inclusion criteria, this cohort of patients, with their (co)prescriptions, reflects the complexity of the real-world system and can be considered suitable for the assessment and validation of methods for the study of drug prescriptions.

### 3.2. Structure of Drug Prescription Networks

**Figure 3:**
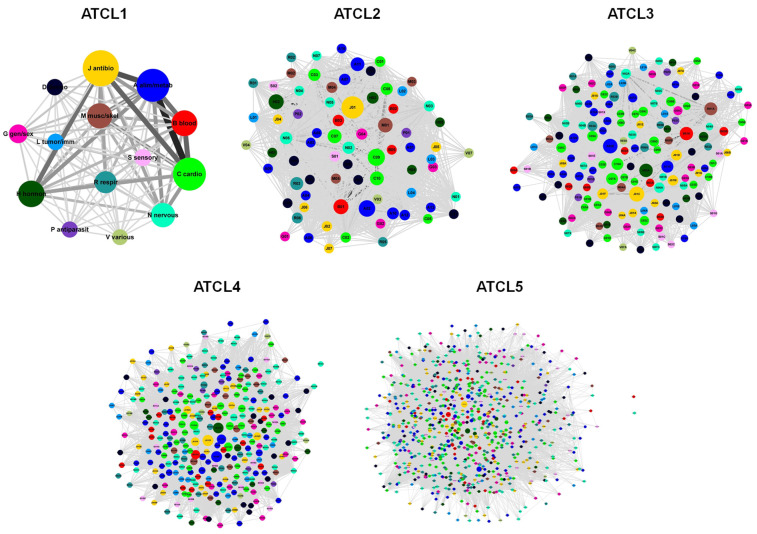
Drug prescription networks. Data on drug prescriptions were analysed to generate five drug prescription networks that correspond to the levels of the ATC classification system (ATCL1–ATCL5). Node diameter and edge thickness were proportional to the number of patients prescribed with the individual ATC*_i_*s and the pairs ATC*_i_*–ATC*_j_* within the ATC level L, respectively. Nodes were coloured according to the 14 groups found at the first level of the ATC classification system.

Pattern recognition in drug prescription datasets is a problem of interest, with relevant potential implications and applications related to prescription behaviour. The adoption of network models, together with network theory tools, may be a means to recognise such patterns. To evaluate this assumption, the structures of the five DPNs (Figure 3) created in this study were analysed. As shown in Figure 3 and Table 1, the number of nodes (N) and edges (E) in the networks increased across the ATC levels. As indicated by the graph density (d), DPN-ATCL1 (14 nodes and 91 edges) was a complete network (a network in which every pair of nodes is connected by a link), whereas DPN-ATCL2–DPN-ATCL5 were progressively more sparse networks, with fewer edges than a complete network.

**Figure 4:**
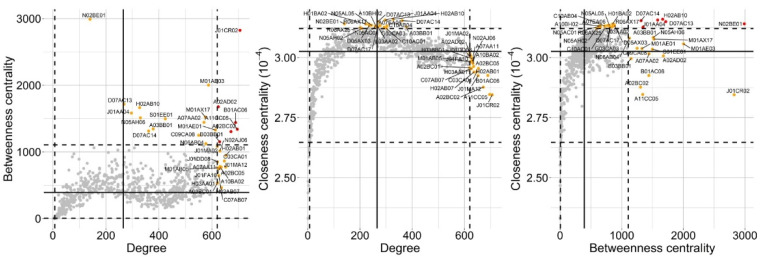
Node degree, betweenness centrality and closeness centrality. To better understand the relative importance of each node (DPN-ATCL5), degree, betweenness centrality and closeness centrality were computed and displayed in the 2D scatter plots. Horizontal and vertical black lines display the 50th (solid line), 2.5th and 97.5th (dashed lines) percentiles. Each node is represented as a dot; coloured (orange and red) dots highlight subsets of ATC codes endowed with the higher relative importance, with respect to the three metrics.

**Figure A3:**
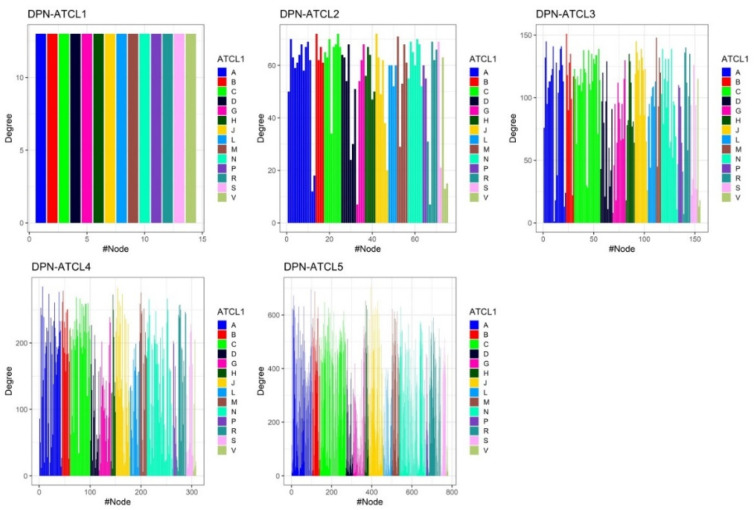
Individual node degree. Data on drug prescription have been represented in the form of DPNs (ATCL1–ATCL5). Individual node degrees were computed and are displayed as bar charts. Nodes are coloured according to the 14 groups found at the first level of the ATC classification system.

Node connectivity was then studied. Each individual node i in a network is characterised by its degree ($k_{i}$), which is the number of links it has with other nodes (referred to as the nearest neighbours) and thereby a “local” quantity. Hence, the degree for a distinct ATC*_i,L_* is the number of pairs ATC*_i,L_*–ATC*_j,L_* (with i≠j) in the DPNs, and can be used as a measure of the variety of co-prescriptions that involve that ATC*_i,L_*. The average degree ($\bar{k}$) increased across the five ATC levels (Table 1). These changes were also accompanied by an increase in the variability of $k_{i}$, which means that both very low-degree and very high-degree ATC*_i,L_* can be found in the DPNs, at the higher ATC levels particularly. As shown in Figure A3, $k_{i}$ ranged from 7 (G01, gynecological anti-infectives and antiseptics, and R02, throat preparations) to 72 (B01, antithrombotic agents, C09, agents that act on the renin–angiotensin system, and J01, anti-bacterials for systemic use) for DPN-ATCL2; from 1 (A07B, intestinal adsorbents) to 151 (B01A, antithrombotic agents) for DNP-ATCL3; from 0 (N07AX, other parasympathomimetics) to 285 (A02BC, proton-pump inhibitors) for DPN-ATCL4; and from 0 (B03XA03, methoxy polyethylene glycol-epoetin beta, and N07AX02, choline alfoscerate) to 706 (J01CR02, amoxicillin and beta-lactamase inhibitor) for DPN-ATCL5. The same degree (13) was determined for all nodes in DPN-ATCL1. Moreover, a giant component exists ($\bar{k}>1$), which contains all of the nodes in the network ($\bar{k}>\ln N$) [8], in almost all of the DPNs. The relative importance of a node i in the DPNs was also estimated by computing two “global” quantities, betweenness centrality ($b_{i}$) and closeness centrality ($Cl_{i}$; Appendix A). As for degree, the average betweenness centrality (${\bar{b}}_{i}$) and the average closeness centrality (${\bar{Cl}}_{i}$) of the DPNs changed across the five ATC levels (Table 1). Moreover, as shown in Figure 4, by adopting these quantities (k, b and Cl), the relative importance of each node in the network can be characterised following distinct perspective. For example, due to its high degree and betweenness centrality, J01CR02 emerged as the most “important” node in the DPN-ATCL5. Moreover, despite the small degree, N02BE01 (paracetamol) was a central node in this network, thanks to its betweenness and closeness centrality.

In order to gain further insights into node connectivity, degree–degree correlation was then quantified by determining the assortativity coefficient, r [13,14]. Negative values were computed for almost all of the DPNs, with an inverse relationship between r and N (Table 1). It can therefore be stated that the DPNs displayed the disassortative mixing of node degrees, which implies that high-degree ATC*_i,L_*s are preferentially linked to low-degree ATC*_j,L_*s.

As suggested by these results, methods based on an analysis of the structural organisation of DPNs may be helpful to the study of patterns in poly-pharmacy.

### 3.3. Structure of Drug Prescription Networks and Popularity of Drug (Co)Prescription

**Figure A4:**
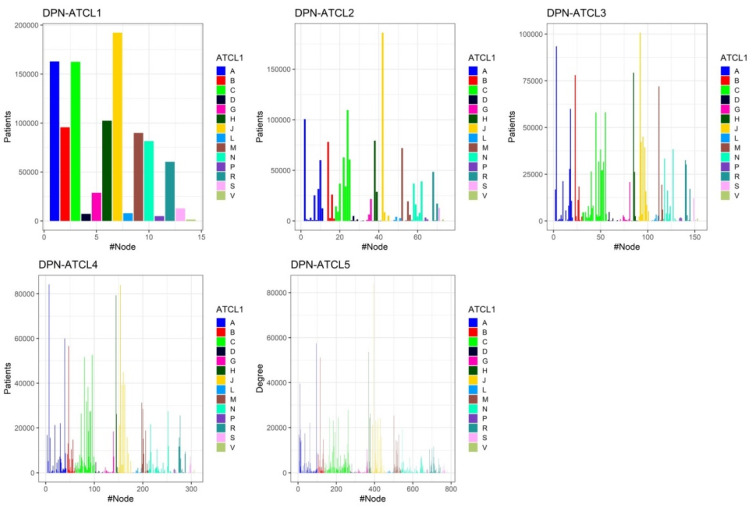
Number of patients prescribed with the ATC*_i_*. Data on drug prescription have been represented in the form of DPNs (ATCL1–ATCL5). The number of patients prescribed with each individual ATC was determined and is displayed in the bar plots. Nodes are coloured according to the 14 groups found at the first level of the ATC classification system.

Previous results that were obtained overlooked the number of patients prescribed with each drug/pairs of drugs, meaning that the “popularity” of each (co)prescription was not considered. However, as the structure of DPNs depends on the proportion of each (co)prescription, it can be hypothesised that conclusions as to the “popularity” of each (co)prescription could also be inferred by investigating the structure of the DPNs. In order to evaluate this hypothesis, the variability associated with drug prescriptions in our study population was first quantified by determining the number of patients prescribed with each ATC*_i_* ($P_{i}$) at the five ATC levels (Figure A4). $P_{i}$ showed wide variety across the ATC levels and ATC codes. Anti-bacterials for systemic use that belonged to the class of penicillins (J, J01, J01C and J01CR02) were found to be the single ATCs that were most commonly prescribed within the first, second, third and fifth ATC levels, respectively. By contrast, larger variability was observed on the opposite side, where many more ATCs (60 at the ATCL5) were prescribed to only one patient over the study period.

**Figure 5:**
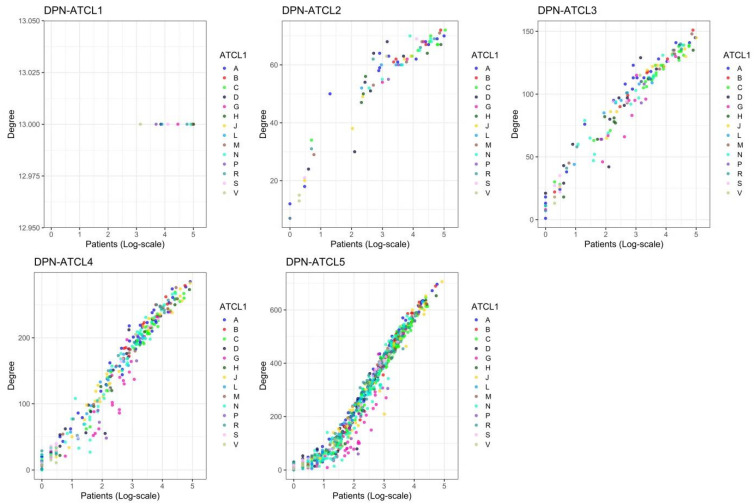
Relationship between number of prescriptions and variety of co-prescriptions. Scatter plots display the relationship between the number of patients prescribed with an ATC*_i_* and the number of neighbours of that ATC*_i_* in the drug prescription networks. Nodes are coloured according to the 14 groups found at the first level of the ATC classification system.

**Figure A5:**
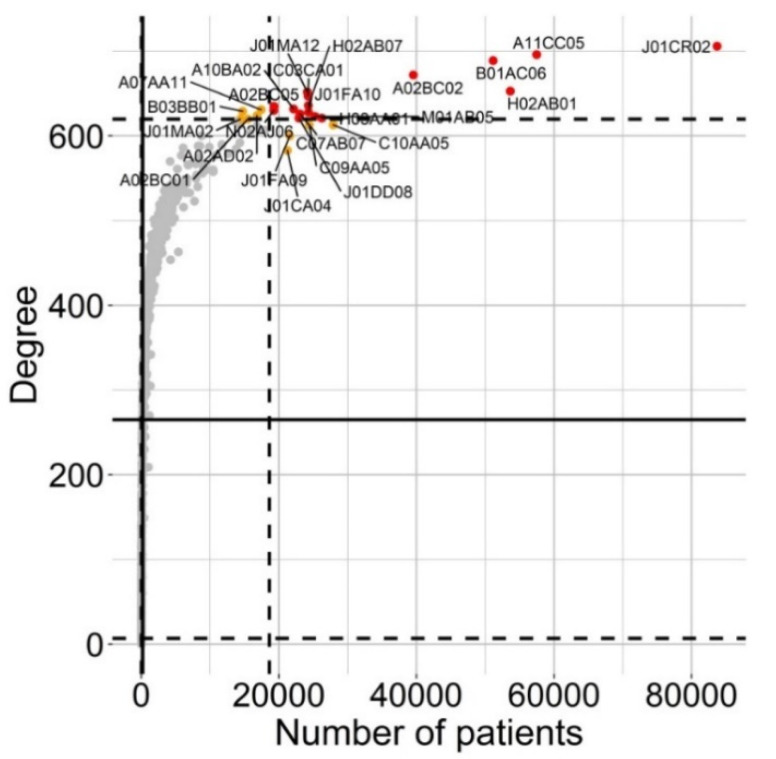
Relationship prescriptions and co-prescriptions in the DPN-ATCL5. The number of patients prescribed with an ATC*_i_* and the number of neighbours of that ATC*_i_* were displayed as a 2D scatter plot. Horizontal and vertical black lines display the 50th (solid line), 2.5th and 97.5th (dashed lines) percentiles. Each node is represented as a dot; coloured (orange and red) dots highlight subsets of ATC codes endowed with the higher values for the two metrics.

The relationship between $P_{i}$ and $k_{i}$ was then studied to assess whether prescriptions and co-prescriptions are related variables. As shown in Figure 5 and Figure A5, the ATC*_i_*s that were more frequently prescribed were also the nodes more connected in the DPNs and *vice versa*, regardless of ATC level. In particular, the most frequently prescribed ATC*_i,L_*s were also the most connected nodes: group J (degree 13), group J01 (72), subgroup J01C (145), subgroup A02BC (degree 285) and chemical substance J01CR02 (degree 706), in DPN-ATCL1, -ATCL2, -ATCL3, -ATCL4, and -ATCL5, respectively. However, some drugs that were prescribed to the same number of patients were co-prescribed together with a different number of other distinct drugs, and this was more likely for the less popular ATCs. For example, in DPN-ATCL5, 13 patients were prescribed with G03GA04 (urofollitropin) and N02BE01 (paracetamol). However, the former was co-prescribed together with 9, while the latter with 140 other distinct drugs.

**Figure 6:**
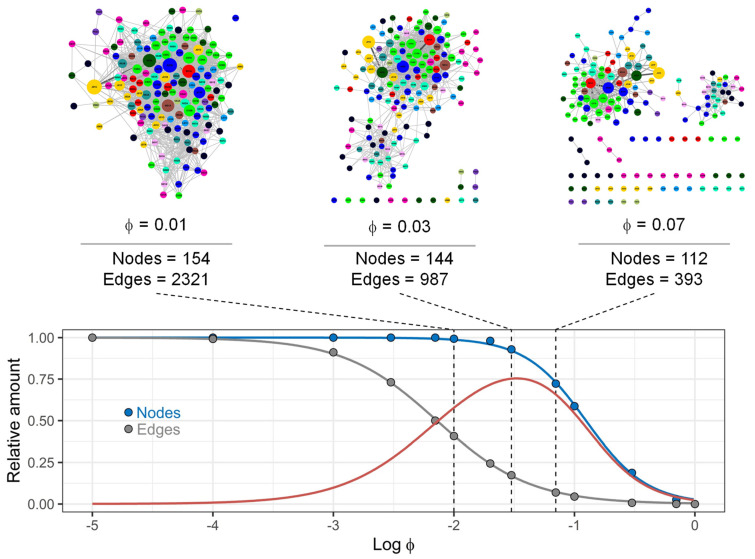
Relationship between *ϕ*-correlation and graph density. Drug prescription networks at the third ATC level were created by adopting different *ϕ*-correlation values (from 0.00001 to 1). The number of connected nodes (blue dots) and edges (grey dots) in these networks was determined and analysed using logistic models (blue and grey lines, respectively). The red curve displays the difference between the blue and the grey curves; the x-coordinate of its maximum is denoted $\phi_{max}$. Nodes are coloured according to the 14 groups found at the first level of the ATC classification system.

**Figure A6:**
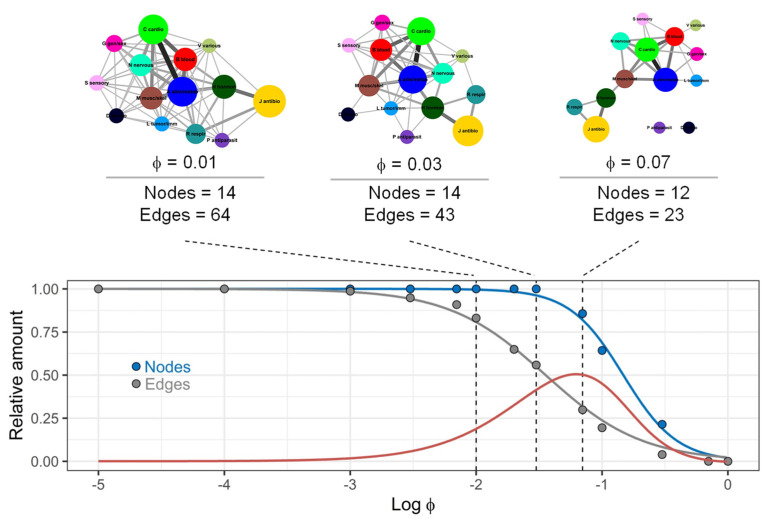
Relationship between *ϕ*-correlation and graph density. Drug prescription networks at the first ATC level were created by adopting different *ϕ*-correlation values (from 0.00001 to 1). The number of connected nodes (blue dots) and edges (grey dotes) in these networks was determined and analysed using logistic models (blue and grey lines, respectively). The red curve displays the difference between the blue and the grey curves; the *x*-coordinate of its maximum is denoted as $\phi_{max}$. Nodes are coloured according to the 14 groups found at the first level of the ATC classification system.

**Figure A7:**
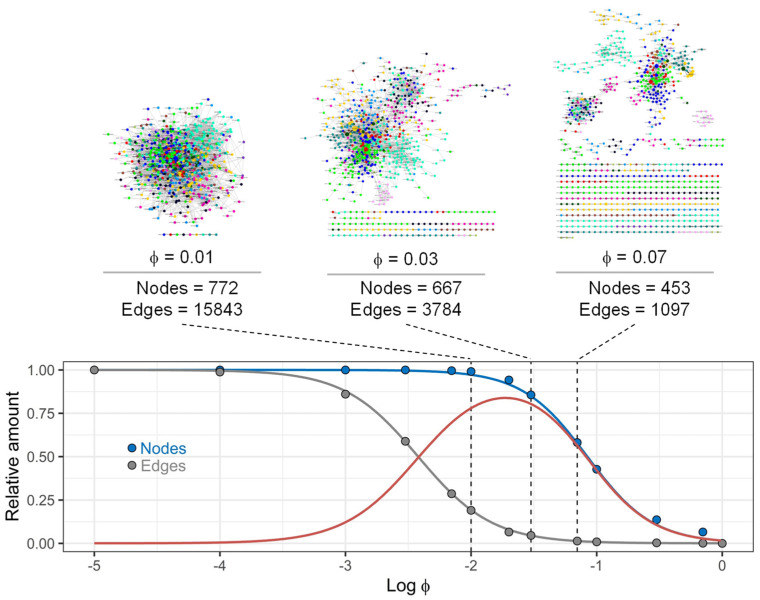
Relationship between *ϕ*-correlation and graph density. Drug prescription networks at the fifth ATC level were created by adopting different *ϕ*-correlation values (from 0.00001 to 1). The number of connected nodes (blue dots) and edges (grey dotes) in these networks was determined and analysed using logistic models (blue and grey lines, respectively). The red curve displays the difference between the blue and the grey curves; the *x*-coordinate of its maximum is denoted as $\phi_{max}$. Nodes are coloured according to the 14 groups found at the first level of the ATC classification system.

Finally, we investigated how, and to what extent, graph density depends on the number of patients (co)prescribed with distinct drugs and drug pairs. The Pearson’s coefficient of correlation $\phi_{ij}$ was calculated for all nodes and edges in the networks [11,15]. Moreover, pre-specified values of $\phi_{ij}$ ($\phi^{*},$ from 0.00001 to 1) were adopted to include (if $\phi_{ij}\geq \phi^{*}$) or exclude (otherwise) nodes and edges. Representative results are shown in Figure 6, Figure A6 and Figure A7. The number of connected nodes and edges in the network decreased in a ϕ-correlation-dependent manner, and these changes were mainly due to the loss of the “weaker” edges, which are edges in the network that are characterised by lower values of $C_{ij}$ compared to the corresponding $P_{i}$ and $P_{j}$. The relative number of connected nodes and edges in the networks at the different $\phi^{*}$ values was also analysed using logistic models. These analyses allowed us to estimate $\phi_{max}$, which was the value of the ϕ-correlation that corresponded to the maximum for the difference between the relative number of connected nodes and edges in the network; these values were 0.063, 0.033 and 0.019 for DPN-ATCL1, -ATCL3 and ATCL5, respectively.

As indicated by these results, the structural organisation of DPNs is strictly dependent on the popularity of drug (co)prescriptions. The structure of DPNs can be manipulated and explored by considering the strength of drug co-prescriptions in order to gain further insight into prescription patterns.

### 3.4. Comparison of Drug Prescription Networks

**Figure 7:**
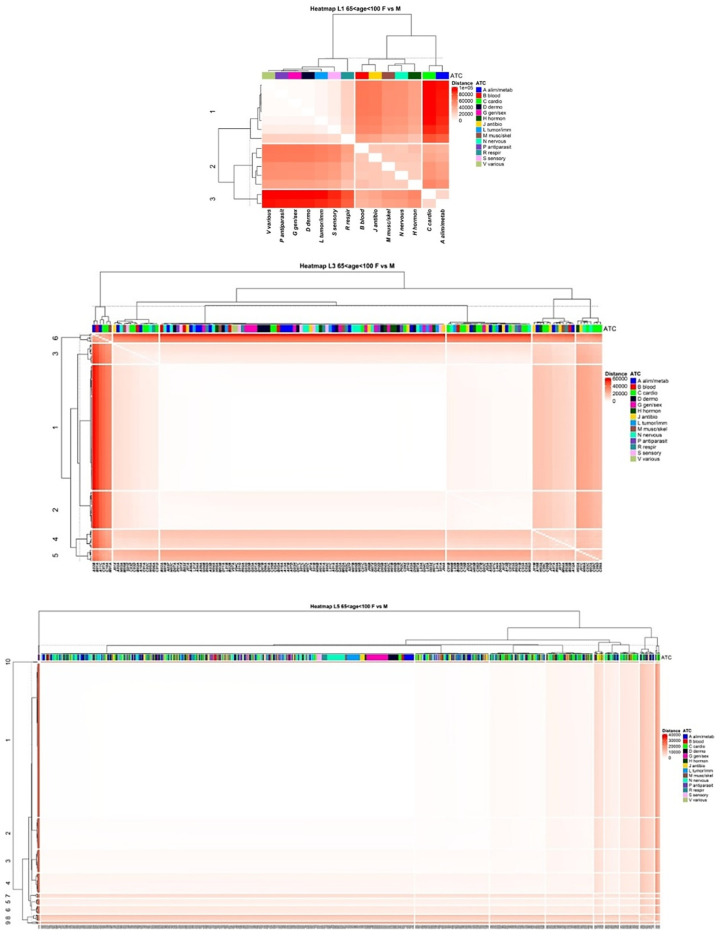
Distance matrices for three ATC levels to compare females vs. males at the 65–100 years-of-age strata. The colour in entry (i, j) is the Euclidean distance between layers i (females) and j (males) of the binary drug prescription networks. Euclidean distances are coded by colours from white (small distance) to red (large distance). Nodes were coloured according to the 14 groups found at the first level of the ATC classification system. Dendrograms identify a number of clusters in the distance matrices.

**Figure A8:**
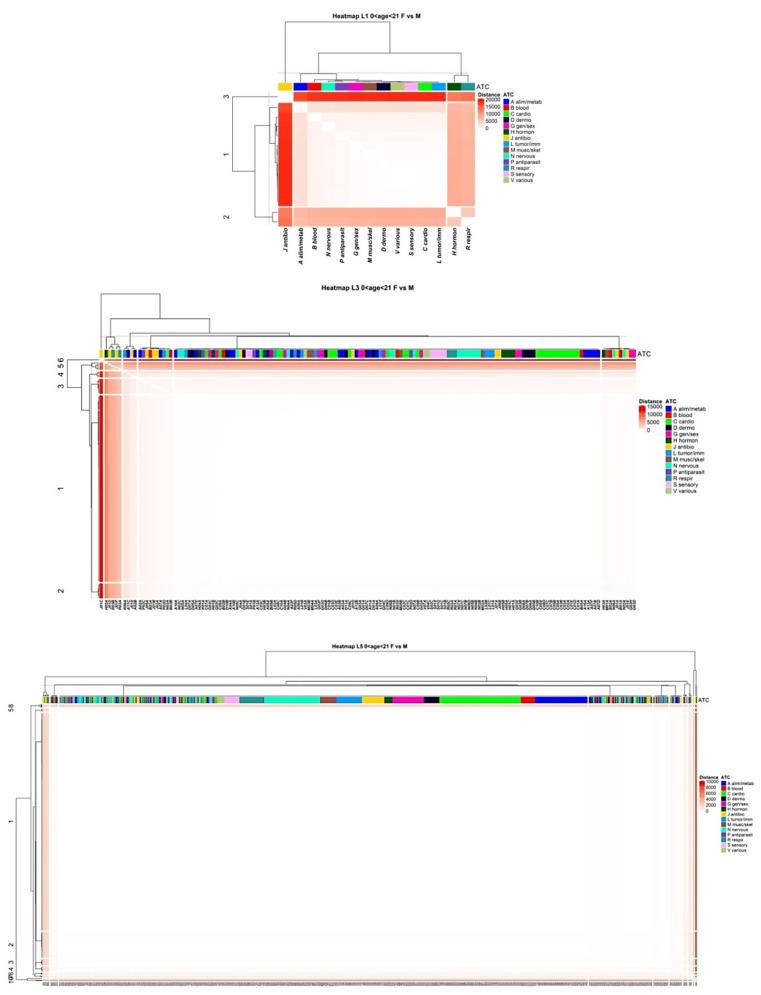
Distance matrices for three ATC levels to compare females vs. males at the 0–21 years age strata. The colour in entry (i, j) is the Euclidean distance between layers i (females) and j (males) of the binary DPN. Euclidean distances are coded by colours from white (small distance) to red (large distance). Nodes are coloured according to the 14 groups found at the first level of the ATC classification system. Dendrograms identify a number of clusters in the distance matrices.

**Figure A9:**
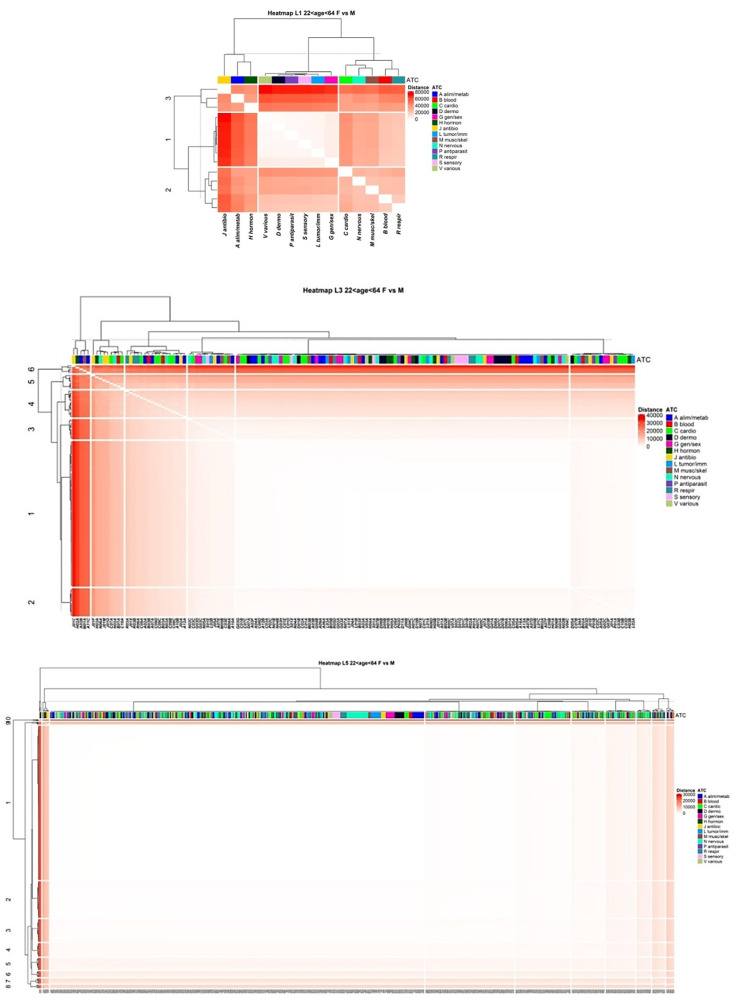
Distance matrices for three ATC levels to compare females vs. males at the 22–64 years age strata. The colour in entry (i, j) is the Euclidean distance between layers i (females) and j (males) of the binary DPN. Euclidean distances are coded by colours from white (small distance) to red (large distance). Nodes are coloured according to the 14 groups found at the first level of the ATC classification system. Dendrograms identify a number of clusters in the distance matrices.

The ability to discover and quantify similarity/difference in distinct (sub)systems is a problem of interest in many research fields. A method based on the computation of distance matrices [16] was therefore used to assess similarity/difference in distinct groups of the study population. The study population was divided into six groups according to gender and age (three strata, 0–21, 22–64 and 65–100 years of age), and data on drug prescriptions were then analysed to give the corresponding DPNs (for ATCL1, ATCL3 and ATCL5). Finally, DPNs were compared by calculating the Euclidean distances between the DPNs created for females and males at the different age-strata and ATC levels. Figure 7 displays examples of 14 × 14, 155 × 155 and 781 × 781 symmetric distance matrices (age strata 65–100 years) for ATCL1, ATCL3 and ATCL5, respectively. Importantly, the presence of a subset of ATCs with comparatively smaller internal distance does not imply the existence of a well-defined group, unless the group itself displays comparatively higher distance to the remaining ATCs. In addition, ATCs were classified by drawing dendrograms, built using the ward.D2 linkage, to facilitate subset identification [17]. Notably, the dendrograms identified a number of clusters in the distance matrices. However, in some cases, they included a large number of ATCs/drugs, while others included only a few ATCs/drugs characterised by a large external distance. For example, cluster 1, in the distance matrix ATCL1, consisted of seven ATCs (R, S, L, D, G, P and V) characterised by the minimal comparative external distance. On the other hand, cluster 3 included only two ATCs (A and C), characterised by the maximal comparative external distance. An even more marked pattern was observed in the distance matrix ATCL5. In particular, cluster 1 included a large number of drugs that belonged to almost all of the ATC groups, whereas cluster 10 included only two drugs, A11CC05 (cholecalciferol) and B01AC06 (acetylsalicylic acid). Additional examples can be found in Figure A8 and Figure A9. Interestingly, comparisons of distance matrices that were computed for the same ATC level, but at different age strata, reveal additional differences (Table A1).

As demonstrated by these results, DPNs provide a means to discover possible differences that may be caused by changes in drug (co)prescriptions over space and time.

## 4. Discussion

The great power of networks to describe real-world systems has attracted growing interest, and this modelling approach has been used in many research areas in recent decades. Several studies have introduced networks as helpful tools to investigate specific questions, even in the field of health and medical information analysis [19,20,21,22]. However, to date, only a few studies have dealt with network models to study drug prescription [10,11,23]. Together with previous findings, our results contribute to the development of the foundations upon which further studies can be designed.

Different types of DPNs (i.e., weighted graphs, bipartite graphs) can be created from the administrative drug prescription records collected by the health systems. Moreover, the structural organisation of these networks and their dynamic changes over time can be investigated to draw conclusions on specific questions of interest and related to the patient and prescriber behaviours, as well as to assess the effects of system-level interventions aimed to rationalise drug prescription (i.e., prescription drug monitoring, clinician and patient education, changes in product labelling and treatment guidelines). For example, the adoption of weighted DPNs to recognise patterns and trends in drug-prescribing data was introduced in previous reports. In particular, Cavallo et al. [10] have considered a set of drug prescriptions (for a total of 42,965 patients) written during a 6-month period by a group of 99 GPs operating in the city of Salerno (Italy). Bazzoni et al. [11] have analysed three datasets (145,072, 186,426 and 171,075 patients, respectively; time window, 1 year) extracted from the administrative prescription database of the Lombardy Region (Italy). In comparison, here we have analysed the largest dataset (5,431,335 entries/drugs prescribed to 361,574 patients over a period of 1 year) to recognise drug prescription patterns. Finally, Hu et al. [23] constructed several bipartite networks with two types of nodes—patients and prescribers—to reveal the characteristics and trends of the interaction between patients and prescribers across Queensland, Australia from 2011 to 2018 (prescribing data for fentanyl patches). Collectively, these examples provide evidence on the potential application of the network approach as a data mining technique, which could support the advisory role of healthcare providers and regulators.

By adopting DPNs, the drug prescription process is studied from the point of view of its topology. Despite the differences in both the original datasets and in the particular kind of interconnection between nodes, DPNs have been consistently demonstrated to be dense graphs [10,11]. Our results extend previous results by showing the relationship between ATC level and graph density. The fact that graph density depends on the relationship between the number of edges and the number of nodes in the network implies that the co-prescription of drugs is a very common event, which can be quantified by specific network measures. In addition, (co)prescription patterns in the original dataset are associated with DPN structure. For example, Newman’s assortative coefficient values reveal that DPNs displayed the disassortative mixing of node degrees, and this property was more pronounced at the higher ATC levels. Therefore, DPNs are characterised by non-random connectivity.

A study of the strength of co-prescriptions has demonstrated that: (*a*) some nodes are linked by weak edges, while others are linked by strong edges; (*b*) communities of densely interconnected nodes that link preferentially to each other than to other nodes in the network (modules) can be recognised by eliminating the weaker edges in the networks. Similarly to previous works [11,15], our aim was to propose a method to gain insights into the modular structure of DPNs after removing the weaker edges. In particular, $\phi_{max}$ can be determined by analysing the following relationships: *ϕ*-correlation vs. number of nodes and *ϕ*-correlation vs. number of edges. This index can be adopted as a threshold value to decrease graph density and focus the analysis toward certain (co)prescriptions selected by means of their “popularity”. Therefore, these results support the conclusion that methods based on the study of DPNs are a flexible approach to the modelling of drug prescription data. Finally, Euclidean distance matrices [16] have been computed in order to discover similarity/difference in multiplex networks.

These analyses, albeit preliminary, have proven to be helpful in quantifying the relative contribution of each ATC in determining gender- and age-specific patterns in drug prescriptions, which likely reflect discrepancies in the prevalence of distinct conditions (e.g., bone disorders). Together with previous analyses, this method may be able to reveal changes in poly-pharmacy patterns over space and time.

Some limits of this study deserve to be considered. The use of DPNs to study the complexity of drug prescription is still in its infancy. Despite the promising results, future studies are needed to better understand the pros and cons. In addition, ASL TO4 does not collect data on drugs not reimbursed by NHS and over-the-counter medications. Moreover, whether the prescribed drugs were really taken is unknown. Therefore, our results cannot provide evidence on the actual drug use.

## 5. Conclusions

There is a growing need to improve our understanding of patterns and trends in drug prescription and utilization. Network models can be used as a data mining technique to study the complexity regarding drug prescription. The results obtained in this study provide further evidence for the efficiency and flexibility that DPNs show when representing drug prescription data. In particular, concepts and metrics of the network theory add novel perspectives in the path from raw data to knowledge, and could provide valuable evidence on how drugs are prescribed.

## Figures and Tables

**Table 1:** Structural and statistical measures of drug prescription networks.

ATCL ^1^	N ^2^	lnN	E ^3^	d ^4^	$\bar{k}$ [CV%] ^5^	$\bar{b}$ [CV%] ^6^	$\bar{Cl}\;(\times \;10^{-4})$ [CV%]	r ^7^
1	14	2.639	91	1.000	13.000 [0.0]	5.571 [374.2]	0.876 [41.5]	NA ^8^
2	75	4.317	2053	0.740	54.747 [32.2]	51.839 [140.4]	50.444 [11.7]	−0.194
3	155	5.043	7191	0.603	92.787 [42.9]	91.999 [104.4]	26.907 [7.9]	−0.189
4	308	5.731	22,747	0.481	147.708 [55.9]	173.130 [85.3]	9.712 [7.2]	−0.264
5	781	6.661	104,063	0.342	266.487 [72.9]	423.840 [74.0]	2.984 [6.4]	−0.332

^1^ ATCL: Anatomical Therapeutic Chemical level; ^2^ N: number of nodes; ^3^ E: number of edges; ^4^ d: network density; ^5^ $\bar{k}$: average degree; ^6^ CV: coefficient of variation (standard deviation/average); ^7^ r: assortativity coefficient; ^8^ NA: not applicable.

## Appendix A

### Appendix A.1. Statistical and Network Measures Adopted in the Study

#### Appendix A.1.1. Degree

The degree of node i in a network $\left(k_{i}\right)$ is the number of nearest neighbours of that node. Moreover, given a network with N nodes, the average node degree of the network is defined by:

$$\bar{k}=\frac{1}{N}\overset{N}{\underset{i=1}{\sum }}k_{i}.$$

Degree quantifies the relative importance of a node in terms of the variety of its connections. It attributes large importance to nodes that are connected to a large number of other nodes in the network [7,15]. In addition, as you need to inspect only one node to find its degree, the degree is a “local” quantity.

#### Appendix A.1.2. Betweenness Centrality

Quantities which need an inspection of the whole network to be computed are “global” in nature—among them, the betweenness centrality. The betweenness centrality of node i in an undirected network $\left(b_{i}\right)$ is given by:

$$b_{i}=\underset{r,s}{\sum }\frac{n_{rs}^{i}}{g_{rs}},$$

where $n_{rs}^{i}$ is the number of shortest paths between node r and node s that pass through node i, and $g_{rs}$ is the total number of shortest paths between node r and node s. The average betweenness centrality of the network ($\bar{b}$) is defined similarly to the average node degree of a network. In general, the betweenness centrality quantifies the relative importance of a node in the communication between other pairs of nodes. In particular, it attributes large importance to nodes that act as bridges between different regions of the network. These are nodes that are traversed by a large number of shortest paths connecting different pairs of nodes in the network. The betweenness centrality is not related to the degree of the node, as nodes of low-degree might connect two large and otherwise unconnected regions of the network and therefore acquire high betweenness centrality [7,15].

#### Appendix A.1.3. Closeness Centrality

The closeness centrality of a node i in an undirected network $\left(Cl_{i}\right)$ is given by:

$$Cl_{i}=\frac{1}{\frac{1}{N-1}\sum_{j}d_{ij}}=\frac{1}{{\bar{l}}_{i}},$$

where ${\bar{l}}_{i}$ is the average distance of the node i to the other nodes in the network. The average closeness centrality of the network ($\bar{Cl}$) is defined similarly to the average node degree of a network. As for the betweenness centrality, even the closeness centrality is a “global” measure, which quantifies the relative importance of a node in a network beyond the number of its connections (nearest neighbours): central nodes are those which are relatively close to all other nodes in the network.

#### Appendix A.1.4. Network Density

The density of a network (d) is defined as follows:

$$d=\frac{2E}{N\left(N-1\right)}=\frac{\bar{k}}{N-1},$$

where E is the number of links and N is the number of nodes in the network. A criterion for considering a network as sparse is that d≪1, which is equivalent to say that the number of nodes is markedly higher than the average node degree of the network [7].

#### Appendix A.1.5. Network Assortativity

It has been shown that in real-world networks nodes are not connected randomly, but instead there is a significant bias for nodes of high degree to connect either to nodes of high degree (assortative networks) or to nodes of low degree (disassortative networks). In these cases, the network displays degree correlations. To establish whether a network is assortative, disassortative or without degree correlations, the assortativity coefficient (r) can be calculated, as proposed by Newman M.E.J. (2002, 2003) [13,14]:

$$r=\underset{k_{i},k_{j}}{\sum }\frac{k_{i}k_{j}\left[E\left(k_{i}k_{j}\right)-Q\left(k_{i}\right)Q\left(k_{j}\right)\right]}{\sigma_{Q}^{2}},$$

where $k_{i}$ and $k_{j}$ are the numbers of neighbours (degree) of node i and j, respectively, minus 1, $E\left(k_{i}k_{j}\right)$ is the fraction of links that connect a node of degree $k_{i}$ to a node of degree $k_{j}.$ Moreover, let $P\left(k_{j}\right)$ be the probability that a node selected at random in the network has degree $k_{j}$, then, the distribution of the excess degree of a node at the end of a randomly chosen link is:

$$Q\left(k_{j}\right)=\frac{\left(k_{j}+1\right)P\left(k_{j}+1\right)}{\sum_{i}k_{i}P\left(k_{i}\right)}.$$

Finally, $\sigma_{Q}^{2}$ is the standard deviation of the distribution $Q\left(k_{j}\right).$ The assortativity coefficient is a number with values between −1 and 1. Negative values of the assortativity coefficient indicate that the network is overall disassortative, while positive values of r indicate that the network is overall assortative [7,15].

#### Appendix A.1.6. The Pearson’s Correlation Coefficient for Binary Variables

The Pearson’s correlation coefficient for binary variables (*ϕ*-correlation or simply *ϕ*) allows the quantification of the strength of co-prescription associations; it is given by:

(A1) $$\phi_{i,j}=\frac{C_{ij}O-P_{i}P_{j}}{\sqrt{P_{i}P_{j}\left(O-P_{i}\right)\left(O-P_{i}\right)}},$$

where $C_{ij}$ is the number of patients co-prescribed with both drugs, O is the total number of patients in the studied population and $P_{i}$ is the prevalence of the $i^{th}$ prescription. When *ϕ* > 0, co-prescription is larger than expected by chance; when *ϕ* < 0, co-prescription is smaller than expected by chance [11,24].

#### Appendix A.1.7. Difference of the Networks (Euclidean Distance)

Given two un-directed networks $G^{\left(1\right)}=\left(V^{\left(1\right)},E^{\left(1\right)},W^{\left(1\right)}\right)$ and $G^{\left(2\right)}=\left(V^{\left(2\right)},E^{\left(2\right)},W^{\left(2\right)}\right)$, where $V^{\left(1\right)}=V^{\left(2\right)}=V$ are finite and identical sets of nodes, $E^{\left(\cdot \right)}$ are finite sets of edges and $W^{\left(\cdot \right)}$ are finite sets of weights, we define the Euclidean distance as:

(A2) $$d_{EUC}=\sqrt{\sum_{i,j\in V}{\left[w_{ij}^{\left(1\right)}-w_{ij}^{\left(2\right)}\right]}^{2}}.$$

### Appendix A.2. The Anatomical Therapeutic Chemical (ATC) Classification System

The ATC classification system is a strict hierarchy, including five levels of classification (L1–L5), and for the anatomical/pharmacological groups (or 1st levels) there are 14 main groups:

A—Alimentary tract and metabolism;
B—Blood and blood forming organs;
C—Cardiovascular system;
D—Dermatologicals;
G—Genito-urinary system and sex hormones;
H—Systemic hormonal preparations, excluding sex hormones and insulins;
J—Anti-infectives for systemic use;
L—Antineoplastic and immunomodulating agents;
M—Musculo-skeletal system;
N—Nervous system;
P—Antiparasitic products, insecticides and repellents;
R—Respiratory system;
S—Sensory organs;
V—Various.
(see https://www.whocc.no/atc/structure_and_principles/, accessed on 25 February 2021).

**Table A1:** Comparisons between DPNs (females vs. males). Representative ATCs endowed with the higher distance values.

ATC	Age Strata (Years)		
	0–21	22–64	65–100
L1	J	J, A and H	A and C
L3	J01C	J01C, H02A, A02B, M01A and A11C	A02B, B01A, A11C, C07A, C10A and M01A
L5	J01CR02 and H02AB01	J01CR02, A11CC05 and H02AB01	A11CC05 and B01AC06
